# Effects of propofol on the inflammatory response during robot-assisted laparoscopic radical prostatectomy: a prospective randomized controlled study

**DOI:** 10.1038/s41598-019-41708-x

**Published:** 2019-03-27

**Authors:** Go Un Roh, Young Song, Junbeom Park, Yu Min Ki, Dong Woo Han

**Affiliations:** 1 Department of Anesthesiology and Pain Medicine, CHA Bundang Medical Center, CHA University School of Medicine, 59 Yatap-ro, Bundang-gu, Seongnami-si, Gyeonggi-do 13496 Korea; 2 Department of Anesthesiology and Pain Medicine, Yonsei University College of Medicine, Gangnam Severance Hospital, 211 Eonju-ro, Gangnam-gu, Seoul 06273 Korea; 3 Anesthesia and Pain Research Institute, Yonsei University College of Medicine, Gangnam Severance Hospital, 211 Eonju-ro, Gangnam-gu, Seoul 06273 Korea

## Abstract

Robot-assisted laparoscopic radical prostatectomy (RALRP) is a minimally invasive procedure; however, some amount of surgical trauma that can trigger systemic inflammation remains. Moreover, pneumoperitoneum during RALRP induces ischemia–reperfusion injury (IRI). Propofol, an anesthetic, is known to have anti-inflammatory and antioxidant properties. In the present study, we compared the effects of propofol with those of desflurane on inflammation and IRI during RALRP via measurements of different biomarkers and evaluation of perioperative renal function. Fifty patients were randomized to receive either desflurane (n = 25) or propofol (n = 25) with remifentanil during RALRP. Serum levels of interleukin (IL)-6 (IL-6), tumor necrosis factor alpha, C-reactive protein, and nitric oxide were measured 10 min after anesthesia induction (T1), 100 min after carbon dioxide (CO_2_) insufflation (T2), and 10 min after CO_2_ deflation (T3). Perioperative urine outputs and the serum creatinine level at 24 h after surgery were also recorded. We found that IL-6 levels at T2 and T3 were higher than those at T1 in both groups, although the increases were significant attenuated only in the propofol group. The other parameters showed no differences among the three time points in both groups. The intraoperative urine output was significantly higher in the propofol group than in the desflurane group, while the creatinine level showed no significant changes in either group. Our findings suggest that propofol can not only attenuate the inflammatory response during and after pneumoperitoneum in patients undergoing RALRP but also prevent oliguria during pneumoperitoneum.

## Introduction

Prostate cancer is currently the most common malignancy in men and the second leading cause of cancer-related death in the West. Over the last decade, minimally invasive radical prostatectomy with robot-assisted laparoscopy has gained popularity for the treatment of prostate cancer^1,2^. Compared with conventional, more invasive procedures, laparoscopic surgery allows for a smaller abdominal incision and causes less tissue trauma along with a reduced stress response. However, such minimally invasive procedures require pneumoperitoneum for adequate visualization and surgical manipulation, and the associated insufflation and deflation procedures often lead to ischemia–reperfusion injury (IRI) and exacerbate inflammation and the oxidative stress response, consequently leading to postoperative complications^3–5^. Although several studies have attempted to minimize inflammation and IRI during laparoscopic surgery, none have reported clinically promising results^6,7^.

Propofol, a popular intravenous agent for the induction and maintenance of anesthesia, is known to have anti-inflammatory and antioxidant effects^8–10^. Previous studies have demonstrated that propofol reduces lipid peroxidation and proinflammatory cytokine levels after myocardial ischemia reperfusion^11^. In a previous study involving craniotomy, propofol was associated with significantly higher anti-inflammatory cytokine levels than was the volatile anesthetic^12^. Furthermore, in an animal model of renal IRI, propofol attenuated oxidative renal damage and accelerated recovery after IRI^13^. On the other hand, desflurane, a common volatile anesthetic, has shown somewhat conflicting results concerning its anti-inflammatory effects. In a mouse model of ventilator-induced lung injury, desflurane did not prevent inflammatory responses and production of reactive oxygen species^14^. Another study found that the increase in inflammatory cytokine levels was lesser with desflurane anesthesia than with propofol anesthesia during coronary artery bypass grafting^15^. However, to our knowledge, no study has evaluated and compared the preventive effects of propofol and desflurane against inflammation and IRI in human patients undergoing robot-assisted laparoscopic surgery.

Therefore, in the present study, we compared the effects of propofol with those of desflurane on inflammation and IRI during robot-assisted laparoscopic radical prostatectomy (RALRP) via measurements of different biomarkers and evaluation of perioperative renal function.

## Results

**Figure 1:**
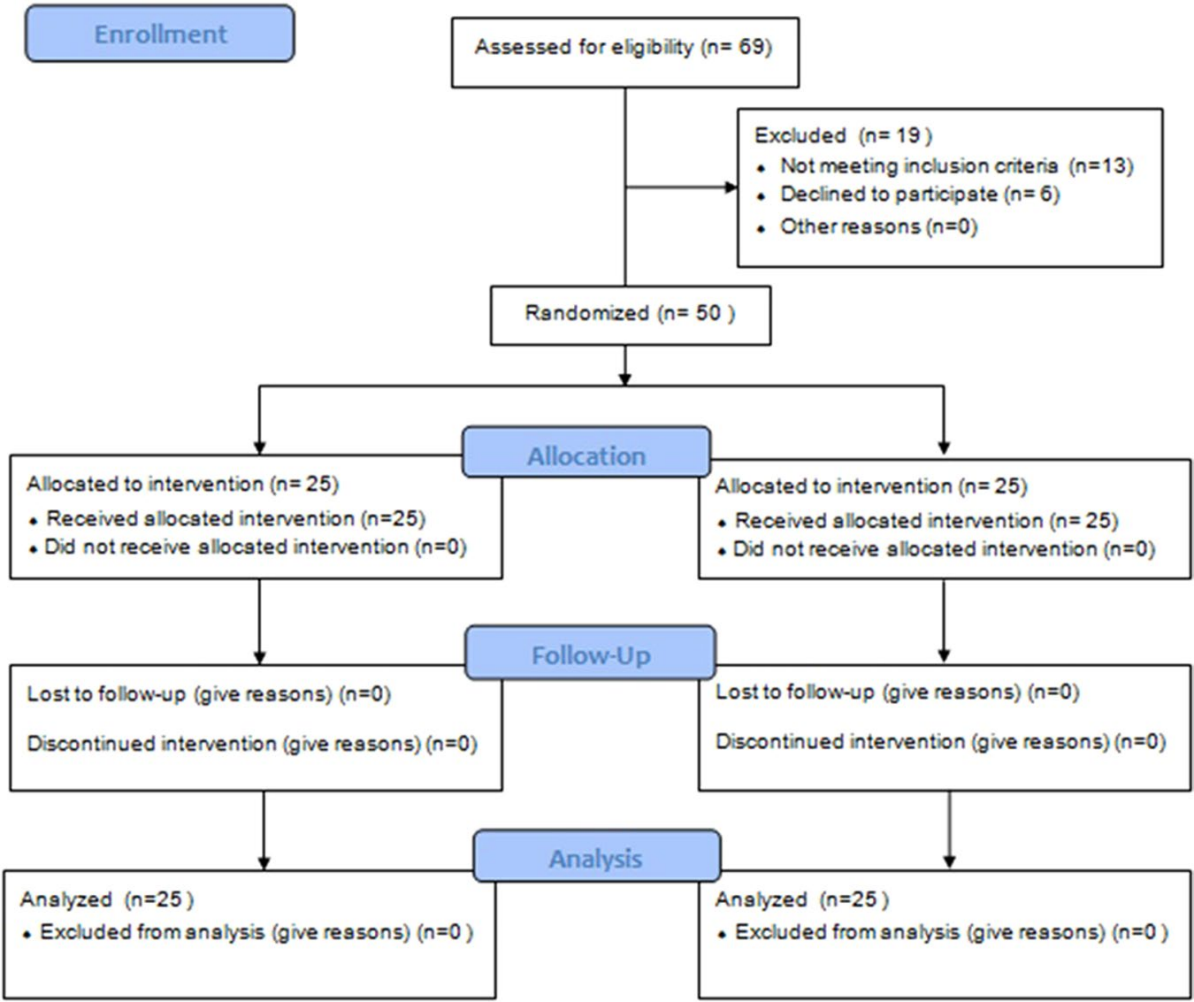
CONSORT diagram showing the participant recruitment process. In total, 50 patients were randomized to receive desflurane (n = 25) or propofol (n = 25) anesthesia during radical robot-assisted laparoscopic radical prostatectomy (RALRP), and all 50 patients completed the study.

All 50 patients completed the study. There were no significant differences in patient characteristics between the propofol and desflurane groups (Fig. 1). Although the number of patients with diabetes mellitus was higher in the propofol group, the difference was not statistically significant (Table 1). The durations of anesthesia, surgery, and pneumoperitoneum were similar between the two groups.

**Table 1:** Characteristics of patients who received propofol or desflurane anesthesia during robot-assisted laparoscopic radical prostatectomy.

	Propofol (n = 25)	Desflurane (n = 25)	p-value
Age (years)	61.7 ± 5.4	63.8 ± 5.0	0.173
Height (cm)	167.8 ± 5.8	168.0 ± 6.1	0.925
Weight (kg)	69.5 ± 6.9	70.0 ± 7.5	0.891
Medical history			
Hypertension	11 (44)	15 (60)	0.258
Diabetes mellitus	6 (24)	1 (4)	0.098
Preoperative medication			
Angiotensin receptor blocker	6 (24)	7 (28)	0.747
Diuretics	2 (8)	3 (12)	1.000

**Figure 2:**
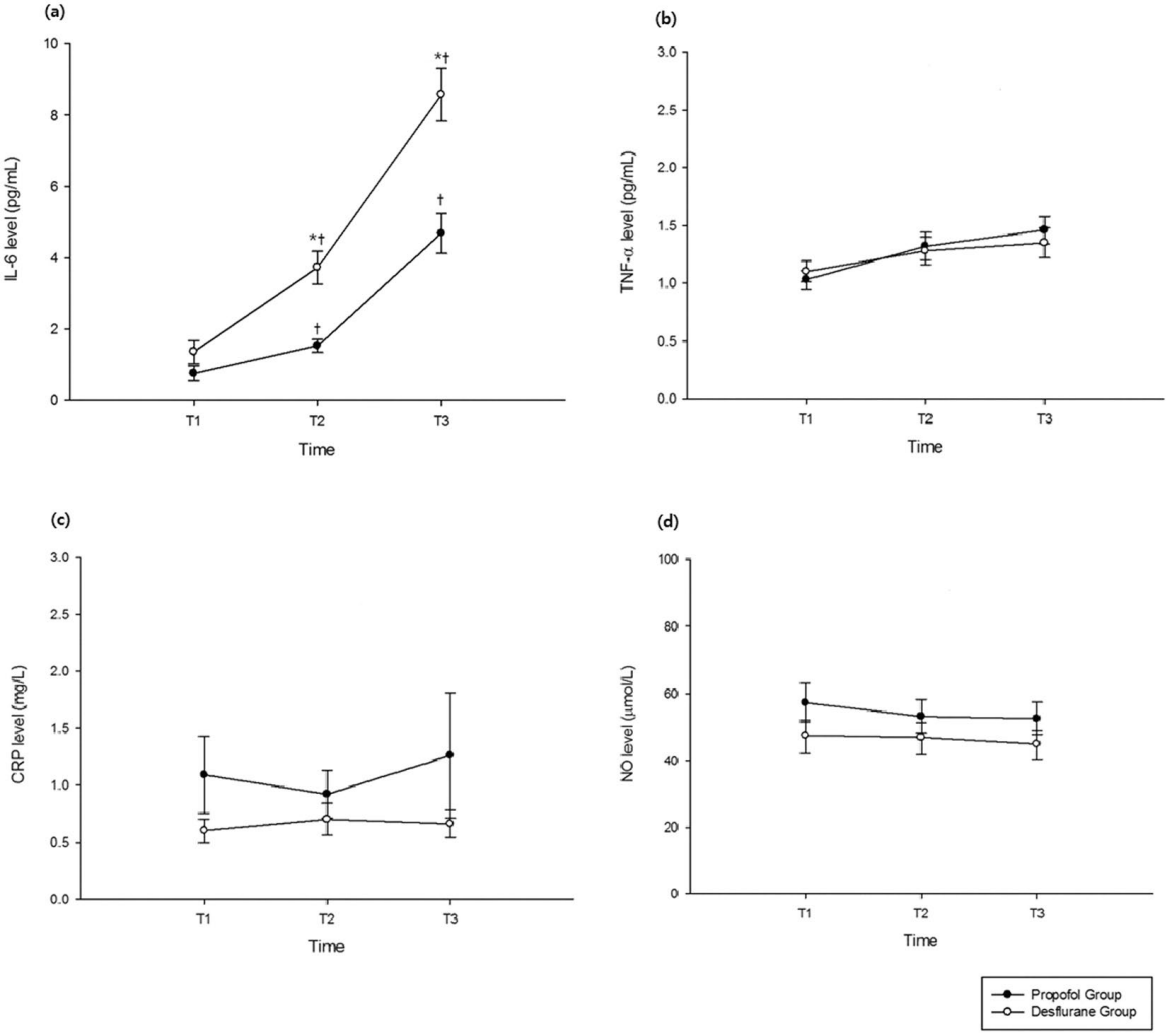
Perioperative changes in interleukin (IL)-6 (**a**), tumor necrosis factor alpha (TNF-α) (**b**), C-reactive protein (CRP) (**c**), and nitric oxide (NO) (**d**) levels in patients who received propofol (n = 25) or desflurane (n = 25) anesthesia during robot-assisted laparoscopic radical prostatectomy (RALRP). T1, 10 min after anesthesia induction; T2, 100 min after pneumoperitoneum; T3, 10 min after carbon dioxide deflation. *p < 0.001 for intergroup comparisons. ^†^p < 0.001 vs. T1.

**Figure 3:**
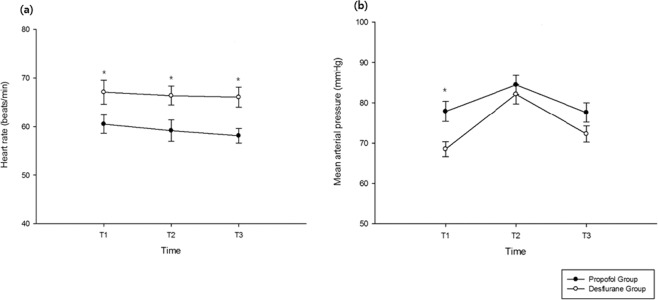
Perioperative hemodynamic changes in the heart rate (**a**) and mean arterial pressure (**b**) in patients who received propofol (n = 25) or desflurane (n = 25) anesthesia during robot-assisted laparoscopic radical prostatectomy (RALRP). T1, 10 min after induction; T2, 100 min after pneumoperitoneum; T3, 10 min after carbon dioxide deflation. *p < 0.05 for intergroup comparisons.

Moreover, IL-6 levels at 10 min after induction (T1) were similar between the two groups. Within groups, IL-6 levels at T2 (p < 0.001) and T3 (p < 0.001) were significantly higher than the level at T1, while intergroup comparisons showed that IL-6 levels at T2 (1.52 ± 0.96 pg/mL vs. 3.72 ± 2.30 pg/mL; p < 0.001) and T3 (4.68 ± 2.76 pg/mL vs. 8.57 ± 3.72 pg/mL; p < 0.001) were significantly lower in the propofol group than in the desflurane group. There were no significant differences in TNF-α, CRP, and NO levels at any time point between the two groups (Fig. 2). The intraoperative urine output was significantly higher in the propofol group (440 ± 235 ml) than in the desflurane group (299 ± 208 ml; p = 0.031), although there were no differences in the amount of fluid intake and intraoperative bleeding. The remifentanil dose was greater in the propofol group (1161 ± 430 mcg) than in the desflurane group (1104 ± 515 mcg; p = 0.001), with no differences in the duration of infusion (Table 2). The heart rate was consistently lower in the propofol group during surgery. The mean arterial pressure (MAP) was comparable between groups at all time points except 10 min after anesthesia induction (77.8 ± 12.3 vs. 68.5 ± 9.3; p = 0.004; Fig. 3). The frequency of hypotension and the required dose of ephedrine were both higher in the desflurane group than in the propofol group (Table 3).

**Table 2:** Intraoperative data for patients who received propofol or desflurane anesthesia during robot-assisted laparoscopic radical prostatectomy.

	Propofol (n = 25)	Desflurane (n = 25)	p-value
Duration of anesthesia (min)	246.8 ± 34.1	242.4 ± 33.4	0.653
Duration of surgery (min)	186.5 ± 32.1	186.0 ± 33.4	0.962
Duration of pneumoperitoneum (min)	147.5 ± 26.9	144.6 ± 25.7	0.697
Fluid intake (mL)	1882.0 ± 483.0	1848.0 ± 619.9	0.830
Bleeding (mL)	329.6 ± 224.0	277.2 ± 191.9	0.379
Urine output (mL)	440.3 ± 234.6	299.0 ± 207.8	**0.031**
Remifentanil			
Dose (mcg)	1611.4 ± 430.0	1103.7 ± 515.2	**0.001**
Dose (mcg/kg/min)	0.10 ± 0.02	0.07 ± 0.02	**<0.001**
Ephedrine			
Patients (number)	7 (28)	18 (72)	**0.002**
Dose (mg)	2.7 ± 5.1	12.6 ± 11.1	**<0.001**

**Table 3:** Perioperative renal profiles for patients who received propofol or desflurane anesthesia during robot-assisted laparoscopic radical prostatectomy.

	Propofol (n = 25)	Desflurane (n = 25)	p-value
Urine output (mL)			
POD 0	1683.7 ± 425.1	1644.2 ± 395.2	0.752
POD 1	2100.4 ± 829.1	1997.9 ± 668.3	0.656
POD 2	2705.2 ± 764.7	2801.4 ± 930.4	0.709
POD 3	2264.4 ± 605.2	2051.4 ± 682.7	0.279
Serum creatinine (mg/dL)			
Before surgery	0.96 ± 0.16	0.93 ± 0.14	0.544
POD 1	0.91 ± 0.15	0.83 ± 0.11	0.057
Difference	−0.05 ± 0.12	−0.10 ± 0.12	0.163

POD: postoperative day.

The postoperative urine output was similar between the two groups until the third postoperative day. Postoperative serum creatinine levels exhibited a similar and clinically insignificant decrease in both groups (Table 3). No patient experienced any surgical or clinical complication, and all were discharged from the hospital in accordance with the guidelines established for their respective surgical procedures.

## Discussion

In the present study, we observed that propofol anesthesia significantly attenuated the increase in IL-6 levels during RALRP, unlike desflurane anesthesia. However, both propofol and desflurane had similar effects on TNF-α, CRP, and NO levels. The intraoperative urine output was significantly greater in the propofol group, although changes in serum creatinine levels were not significantly different between the two groups.

Compared with conventional open surgery, laparoscopic surgery is associated with smaller incisions, reduced intraoperative bleeding, and lesser postoperative pain; however, it may result in increased trauma due to peritoneal insufflation and increased intra-abdominal pressure. In addition, hemodynamic changes and the presence of CO_2_ may further contribute to inflammation and oxidative stress during pneumoperitoneum, which result in acute-phase responses such as the release of proinflammatory cytokines and acute-phase proteins^16–20^. Postoperative increases in inflammatory markers, including cytokines and CRP, are associated with tissue damage, postoperative morbidity, and cancer recurrence^21–23^. However, despite the high number of patients undergoing laparoscopic or robotic surgery, few comparative urology-related studies have investigated inflammation induced by surgical tissue damage or pneumoperitoneum-related IRI^2,24^.

IL-6 is one of the most important ILs that is regulated by both surgery and anesthesia. It is considered the most sensitive marker of the inflammatory response to tissue damage^2^. Among the inflammatory cytokines investigated in our study, IL-6 was significantly increased after surgery in both groups. This finding suggests that IL-6 may represent an appropriate marker of surgical stress in patients undergoing radical prostatectomy for prostate cancer, consistent with the findings of several previous studies^2,25,26^. Because elevated serum IL-6 levels are associated with the aggressiveness of prostate cancer and may represent a predictive marker of biochemical recurrence after prostatectomy, preoperative evaluation of changes in serum IL-6 levels may be valuable for not only surgical stress detection but also prognosis prediction^21^. Compared with the increases in IL-6 levels in the desflurane group, those in the propofol group were significantly attenuated during CO_2_ insufflation and after the termination of pneumoperitoneum in the present study. This result can be attributed to the anti-inflammatory properties of propofol, which have been addressed in numerous studies. Clinically relevant concentrations of propofol inhibit the functions of neutrophils, which include chemotaxis, phagocytosis, and production of reactive oxygen species^27^. In a model of sepsis involving lipopolysaccharide-activated macrophages, propofol was shown to inhibit the production of IL-6 by macrophages by 83%^10^. Furthermore, in clinical settings, propofol significantly lowers IL-6 production following reperfusion during cardiopulmonary bypass^28,29^. Similarly, in a study comparing propofol and sevoflurane anesthesia, the former significantly decreased neutrophil infiltration and systemic inflammation during aortic surgery^23^.

Although the mechanism underlying these effects remains unclear, several reports have suggested that they may be associated with intracellular calcium signaling. According to a report by Tang *et al*., propofol promotes the expression of annexin A1 (a membrane calcium protein), which negatively regulates the activation of the p38 signaling pathway in the mitogen-activated protein kinase system and consequently inhibits the release of inflammatory cytokines such as IL-6^30^. Yang *et al*. further reported that propofol suppresses formyl peptide receptor 1-induced human neutrophil activation via complete blockade of calcium, AKT, and ERK1/2 signaling^31^.

In the present study, there were no changes in serum TNF-α and CRP levels at any time point in both groups. Narita *et al*. reported no increases in TNF-α levels during laparoscopic radical prostatectomy, consistent with our findings, although such increases were observed during open radical prostatectomy^22^. Different inflammatory conditions result in different patterns of alteration in the levels of various stress markers^32^. Our results suggest that the TNF-α level may not be a useful marker of stress in patients undergoing laparoscopic procedures such as RALRP. Furthermore, no significant changes in CRP levels were observed in either group. Similarly, a previous study reported that CRP levels remain steady during laparoscopic surgery but increase during the postoperative period^2^. However, further studies are required to determine whether the changes observed in our study persist after RALRP.

There is a strong association between inflammation and oxidative stress, which interact to produce adverse events in several conditions. Free radicals and reactive oxygen species are risk factors for chronic inflammation and exhibit significantly increased expression during the inflammatory response^33^. Oxidative stress can by quantified by measurement of various biomarkers such as NO and malondialdehyde. Immediately after ischemia, NO levels decrease and vasoconstriction occurs. Subsequently, after reperfusion, NO levels increase because of increased activity of iNOS; this can contribute to the development of problems such as renal injury and dysfunction^34,35^. However, in the present study, NO levels showed no significant changes at any time point, which suggests that renal ischemia did not occur during pneumoperitoneum, and that reperfusion injury after desufflation may not be strong enough to increase iNOS activity. One possible explanation for this finding is that the CO_2_ pressure was maintained at <15 mmHg during surgery; this pressure is reported to have a relatively small effect on NO production, although this finding remains debatable^36^.

Research has demonstrated that pneumoperitoneum may induce transient physiological changes in the kidney, resulting in functional and structural damage associated with transient increases in creatinine values and a decrease in urine output^37^. The conventionally used CO_2_ pressure of 15 mmHg has been reported to decrease renal blood flow by 25%^38^. Oliguria during pneumoperitoneum can be improved by hydration, maintenance of optimal hemodynamic parameters, proper positioning, and administration of protective agents such as N-acetylcysteine or zinc. However, the protective effects of these methods remain controversial, and the mechanisms underlying such effects have not been determined^39–41^. In the present study, the intraoperative urine output was significantly greater in patients who received propofol than in those who received desflurane, although there were no differences in the amount of intraoperative fluid intake and bleeding. These findings can be attributed to the anti-inflammatory and antioxidant effects of propofol. This theory is supported by the findings of a previous study involving patients undergoing mechanical ventilation, where the urine output increased in participants receiving propofol^42,43^. In an animal model of renal IRI, it was shown that propofol mitigated systemic inflammation and tubular damage in the kidney^44^. In other studies of valvular heart surgery, the incidence of acute kidney injury was significantly lowered in patients who received propofol than in those who received a volatile anesthetic^45,46^. Similarly, the anti-inflammatory effects of propofol, which were represented by the decreased IL-6 levels in the present study, may have increased the intraoperative urine output. It is also possible that these effects were due to the maintenance of more stable hemodynamic states during surgery under propofol anesthesia. Patients receiving propofol anesthesia in our study required significantly lower doses of inotropic agents, although previous studies have reported that propofol causes a profound decrease in the systemic blood pressure^41^. Another possible explanation is that remifentanil, which has shown anti-inflammatory and antioxidant effects in *in vitro* and *in vivo* studies^47,48^, may have contributed to the prevention of IRI. The amount of remifentanil infused in the propofol group was significantly greater than that infused in the desflurane group, and this may have increased the intraoperative urine output. Although the renoprotective effects of remifentanil have been investigated at various doses, a dose of 0.1–2 mcg/kg/min was found to exhibit renoprotective effects in previous studies of IRI^49–51^. Accordingly, the doses of remifentanil used in the propofol (0.1 mcg/kg/min) and desflurane (0.07 mcg/kg/min) groups in the present study were somewhat lower than the effective dose affecting stress hormones and inflammatory responses. Further studies should investigate this topic in detail. On the other hand, the postoperative urine output and creatinine levels at 24 h after surgery showed no significant differences between the two groups in the present study. However, this could be critical in cases of limited renal functional reserve, even if the renal blood flow and function return to normal after CO_2_ deflation^41,52^. In fact, recent clinical studies of major noncardiac surgeries have reported a close association between intraoperative oliguria and postoperative renal compromise^53,54^. Further studies are required to clarify the effects of propofol and volatile anesthesia on postoperative clinical outcomes in various surgical cohorts.

This study has several limitations. First, only intraoperative serological markers were analyzed. As previously mentioned, IRI-induced inflammatory responses may persist until 24 h after surgery^2,34^. In addition, postoperative outcomes except those related to the kidney were not evaluated. Further studies are required to elucidate the effect of propofol on inflammatory responses in later postoperative stages. Second, CRP and NO levels were consistently higher in the propofol group, although the differences were not statistically significant. We believe that an increase in the sample size could lead to statistically significant differences. However, CRP and NO levels in the study were mostly within the reference range, so it can be considered that the influence of pneumoperitoneum and anesthetic type on CRP and NO levels during RALRP are not as drastic as believed.

In conclusion, the findings of this study suggest that propofol anesthesia suppresses the inflammatory response during and after pneumoperitoneum and improves the intraoperative urine output in patients undergoing RALRP.

## Methods

### Study design

The present study was approved by the Institutional Review Board of Yonsei University Gangnam Severance Hospital (3-2013-0098) and registered at http://clinicaltrials.gov (NCT02149628, registered on May 29, 2014). Informed consent was obtained from all patients prior to their participation in the study.

Patients (age range: 20–70 years, American Society of Anesthesiologists class I or II) scheduled for RALRP at Yonsei University Gangnam Severance Hospital between July 2014 and July 2015 were included in this study. Patients with renal failure (estimated glomerular filtration rate, <60 ml/min/1.73 m^2^), obesity (body mass index, >30 kg/m^2^), allergies to propofol or peanuts, and/or inability to read were excluded^55^. The enrolled patients were randomly allocated (1:1) to a propofol group or a desflurane group using a randomization table prepared using a random sequence generator (www.random.org). All procedures and measurements were performed in accordance with the relevant guidelines and regulations of our institute.

Before surgery, all patients received intravenous midazolam (0.02 mg/kg) and glycopyrrolate (0.004 mg/kg), following which standard monitoring devices (noninvasive blood pressure, pulse oximetry, electrocardiography, and bispectral spectrometry) were applied. In the propofol group, anesthesia was induced with intravenous propofol [Schnider model with effect site concentration (Ce) of 3–4 mcg/ml] and remifentanil (Minto model with Ce of 3–4 ng/ml) administered via target-controlled infusion (TCI; Orchestra^TM^ BasePrimea, FreseniusVial, France). In the desflurane group, anesthesia was induced with 4 mg/kg of thiopental sodium and remifentanil administered via TCI (Minto model with Ce of 3–4 ng/ml). When the bispectral index (BIS) decreased to <60, rocuronium (0.6 mg/kg) was administered prior to tracheal intubation, following which the patients were mechanically ventilated with a 50% oxygen-in-air mixture. The ventilator was adjusted to maintain a peak airway pressure of <35 cm H_2_O (tidal volume, 7–8 mL/kg of the ideal body weight for volume-controlled ventilation). The respiratory rate was adjusted to maintain an end-tidal carbon dioxide (ETCO_2_) pressure of 40 ± 3 mmHg. TCI of remifentanil (Ce of 1–5 ng/ml) was used for anesthesia maintenance in both groups, with BIS maintained between 40 and 60. Following the induction of anesthesia, the radial artery was cannulated for invasive blood pressure monitoring. Atropine (0.5 mg) was injected when the patient’s heart rate decreased to <50 beats/min. Hypotension was defined by a systolic blood pressure of <90 mmHg or a mean blood pressure of <60 mmHg, and it was treated by 4 mg of ephedrine with 200 ml of crystalloid. During the RALRP procedure, the abdominal cavity was insufflated with carbon dioxide (CO_2_) at a pressure of 15 mmHg, following which the patients were placed in a 30° Trendelenburg position for surgery. When the surgery neared completion, the patients were returned to the supine position, the abdominal cavity was deflated, and an incision measuring approximately 5 cm was placed for specimen removal.

Blood samples were collected through the radial arterial line at 10 min after induction (T1), 100 min after pneumoperitoneum (T2), and 10 min after CO_2_ deflation (T3). Subsequently, the samples were centrifuged (14,000 rpm, 15 min) and the separated serum was stored at −80 °C until analysis^56^.

### Analysis of biomarkers

Biomarkers were assayed in the biochemical laboratory of our institution after study completion. An ELISA kit (Quantikine^®^, R&D System Inc., Minneapolis, MN, USA) was used to assess the levels of interleukin (IL)-6 (IL-6) and tumor necrosis factor alpha (TNF-α). The samples and reference standards were placed into the wells of microplates coated with monoclonal antibodies for each cytokine. The plates were washed to remove nonadherent materials, following which enzyme-linked polyclonal antibodies specific for each cytokine were added to each well. After washing for the removal of nonadherent antibody–enzyme material, substrate solution and amplifier solution were added for color development. After the stop solution was added, absorbance was measured on a plate reader (SpectraMax 190 ELISA Reader, Molecular Devices, China).

### C-reactive protein (CRP)

The latex agglutination method was used for the measurement of CRP levels (N-Assay LA CRP-S D-TYPE, Nittobo, Japan). Latex particles saturated with anti-CRP antibody were added to the sample to induce aggregation of particles via antigen–antibody reactions. The change in absorbance was measured at a wavelength of 572 mm (Hitachi 7600-10, Hitachi, Japan), which was proportional to the concentration of CRP in the sample, which was calculated on the basis of interpolation with the standard curve.

### Nitric oxide (NO)

Levels of NO were determined through the measurement of nitrite levels using an ELISA kit (Parameter^®^ Total Nitric Oxide and Nitrate/Nitrite Assay, R&D System Inc.). We first measured the nitrite concentration (X) in the sample. Subsequently, reductase was added for conversion from nitrate to nitrite, and the total nitrite concentration (Y) was measured. The final nitrate concentration was calculated as the difference between X and Y. Absorbance was measured on a plate reader (SpectraMax 190 ELISA Reader, Molecular Devices).

### Study end points

The serum levels of IL-6, TNF-α, CRP, and NO were considered the primary endpoints, while the perioperative urine outputs and serum creatinine levels were considered secondary endpoints.

### Statistical analysis

Based on a report by Ozmen *et al*., we determined that 25 patients per group were required for the detection of a 20% decrease in IL-6 levels with a power of 80% and type I error of 0.05^57^. SPSS version 18.0 (SPSS Inc., Chicago, IL, USA) was used for all statistical analyses. Variables are presented as means with standard deviations or numbers with percentages, as appropriate. Continuous variables were evaluated using Student’s *t*-tests or Mann–Whitney U-tests, while categorical variables were evaluated using chi-square tests or Fisher’s exact tests. The Bonferroni method was used for multiple comparisons. According to this method, a two-sided p-value of <0.0004 (=0.05/12) indicated statistical significance for the primary end points. For the other parameters, a two-sided p-value of <0.05 was considered statistically significant.

## Supplementary information

Study Protocol

Study Data

## Data Availability

All data generated or analyzed during this study are included in this published article and its Supplementary Information files.
