# Supplementary material for: Effects of propofol on the inflammatory response during robot-assisted laparoscopic radical prostatectomy: a prospective randomized controlled study
Source: Sci Rep. 2019 Mar 27;9:5242. doi: 10.1038/s41598-019-41708-x (PMC6437140; doi:10.1038/s41598-019-41708-x)
Supplement: Supplementary file 1 — Study Protocol [file 41598_2019_41708_MOESM1_ESM.docx]

**Project summary**

Robot-assisted laparoscopic radical prostatectomy (RALRP) has gained popularity because of the expected clinical advantages of minimally invasive procedures. However, there is still remaining surgical trauma to trigger systemic inflammatory response. In addition, insufflation and deflation of pneumoperitoneum during laparoscopic surgery may result in ischemia-reperfusion injury (IRI) of internal organs. The anesthetic agent propofol is known to have anti-inflammatory and antioxidant properties. In the present study, we compared the effects of propofol and desflurane on inflammation and IRI during RALRP by measuring levels of serologic markers, including interleukin 6 (IL-6), tumor necrosis factor alpha (TNF-α), C-reactive protein (CRP), and nitric oxide (NO). We also compared postoperative renal function.

Fifty patients scheduled for RALRP were allocated into two equal groups to receive either desflurane (n=25) or propofol (n=25) in conjunction with remifentanil. Anesthetic agents were adjusted to maintain a bispectral index of 40-60. Remifentanil was adjusted to the degree of surgical stress. Serum levels of IL-6, TNF-α, CRP, and NO were measured 10 min after anesthesia induction (T1), 100 min after CO_2_ insufflation (T2), and 10 min after CO_2_ deflation (T3). Intraoperative and postoperative urine outputs were also recorded, while serum creatinine was measured 24 h after surgery.

It is expected that propofol reduce the inflammatory response and oxidative stress during and after pneumoperitoneum, which could affect the postoperative outcomes.

Influence of propofol on oxidative stress and inflammation during laparoscopic surgery

(NCT02149628, registered at May 29, 2014)

Corresponding Author: Dong Woo Han, MD, PhD

Department of Anaesthesiology and Pain Medicine and Anaesthesia and Pain Research Institute, Yonsei University College of Medicine, Gangnam Severance Hospital, 211 Eonju-ro, Gangnam-gu, Seoul 06273, Korea.

Tel: 82-2-2019-3520

Fax: 82-2-3463-0940

E-mail: [hanesth@yuhs.ac](mailto:hanesth@yuhs.ac)

Funding information: This study was supported by a faculty research grant of Yonsei University College of Medicine for 2013 (6-2013-0098), 50-1, Yonsei-ro, Seodaemun-gu, Seoul 03722, Korea

**Rationale and background information**

During laparoscopic, pneumoperitoneum with pressure of 12-15 mmHg is applied for better visualization for surgery, which results in ischemia of internal organs and ischemia-reperfusion injury (IRI). IRI induces hypoxia, reoxygenation, increases of oxidative stress and acceleration of inflammatory reaction related to endothelial activation. In addition, nitric oxide (NO) also gets attention because the decrease of NO activity during laparoscopic surgery is known to be associated with ischemia of internal organs. There has been constant effort to reduce the IRI during laparoscopic surgery including calcium channel blocker, anti-oxidant, renin-angiotensin inhibitor, TNF-α antibody and anti-inflammatory drugs, but there are not enough clinical studies yet.

Propofol is known to act as a scavenger of reactive oxygen species in animal and in vitro studies, decrease lipid peroxidation in liver, kidney, heart and lung, reduce the production of TNF-α and IL-6. In addition, propofol reduces the synthesis of iNOS after IRI, increases the activity of eNOS, and prevents NF-κB activation, which protects tissue from imflammation responses after IRI. However, most of the studies were limited in animal or in vitro studies and clinical studies were usually limited in cardiac or transplantation surgeries. Therefore, in this study, the influences of propofol on inflammatory reaction and NO production during robot-assisted laparoscopic radical prostatectomy (RALRP) are compared to that of desflurane.

**Study goals and objectives**

The goal of present study is to compare the influence of propofol on inflammatory response and NO production with that of desflurane during RALRP. As markers for inflammatory responses, IL-6, TNF-α, CRP will be measured. NO will be measured. In addition, as secondary end points, influence of propofol on kidney function will be also compared to that of desflurane.

**Study Design**

This randomized controlled study was carried out in Yonsei University Gangnam Severance Hospital operating room during 2 years after IRB approval. Informed consent was obtained from all patients prior to their participation in the study, which included fifty patients (age range: 20-70 years, American Society of Anesthesiologists class I or II) scheduled for RALRP at Yonsei University Gangnam Severance Hospital between July 2014 and July 2015. Patients with renal failure (estimated glomerular filtration rate less than 60 ml/min/1.73 m^2^), allergies to propofol or peanuts, and those who could not read were excluded ^42^. Patients were randomly allocated (1:1) to either a propofol group or desflurane group using a randomization table originated from a random sequence generator at [www.random.org](http://www.random.org). Based on the previous report, we determined that 25 patients (including 10% withdrawal during study) will be required in each group to detect a 20% decrease in levels of IL-6 with a power of 80% and type I error of 0.05.

**Methodology**

After obtaining of informed consent from patients scheduled for RALRP the day before surgery, patients will be randomly allocated either desflurane (n=25) or propofol (n=25) group according to the randomization table generated from [www.random.org](http://www.random.org). Allocation and anesthesia of the study patients will be performed by the anesthesiologist not involved in the study. Study patients, the surgeon and the anesthesiologist involved in the study were blinded to allocation. Patients will be premedicated with midazolam 0.02 mg/kg, glycopyrrolate 0.004 mg/kg in preanesethesia care unit. After standard monitoring including EKG, noninvasive blood pressure, pulse oximetry and bispectral index, anesthesia will be induced with either 2.5% thiopental sodium 4 mg/kg and remifentanil target controlled infusion (TCI, Orchestra^TM^ BasePrimea, FreseniusVial, France) using Minto model or propofol TCI using Schnider model and remifentanil TCI according to allocated group. After the level of BIS below 60, rocuronium 0.6 mg/kg will be injected for intubation. For maintenance, either desflurane or propofol TCI will be used with oxygen-air mixture guided by BIS 40-60. Tidal volume will be set as 8 ml/kg of ideal body weight and respiratory rate will be adjusted to maintain end-tidal carbon dioxide value 40±3 mmHg. After induction of anesthesia, radial artery will be cannulated with 20 gauge catheter. All management except anesthetic agent will be identical in every patient.

Blood sampling was performed at 10 min after anesthesia induction (T1), 100 min after pneumoperitoneum (T2) and 10 min after deflation of pneumoperitoneum (T3) for serum IL-6, CRP, TFN-α and NO.

Serum creatinine was measured at 24 postoperatively. Urine outputs at POD 1, 2 and 3 were recorded.

Intraopertive parameters including mean arterial blood pressure, heart rate, fluid intake, urine output, bleeding, hypotension, use of ephedrine (dose and frequency) and duration of anesthesia, operation and pneumoperitoneum were recorded.

If heart rate dropped below 50/min, 0.5 mg of atropine was injected. Either mean arterial blood pressure was lower than 60 mmHg or systolic blood pressure was lower than 90 mmHg or decreased more than 20 % of baseline, ephedrine 4 mg was injected.

**Safety Considerations**

The anesthetic agents used in this study are commonly used clinical anesthetic practice and the incidence of side effects or complication during this study will be similar to other clinical anesthetic practices in this institution. There will be no difference in anesthetic technique between study patients and others and there will be no need to report additional risk or safety issue related to this study. However, if there are any unpredictable events related to anesthesia occurred during study, the study will be immediately stopped and the detail will be reported to IRB.

In addition, if the patients or guardians refuse to proceed during follow up postoperatively, study will be immediately stopped and the patients will be excluded.

**Follow-up**

The anesthetic agents used in this study are commonly used clinical anesthetic practice and the incidence of side effects or complication during this study will be similar to other clinical anesthetic practices in this institution. There will be no difference in anesthetic technique between study patients and others and there will be no need to report additional risk or safety issue related to this study. However, if there are any unpredictable events related to anesthesia occurred during study, the study will be immediately stopped and the detail will be reported to IRB.

In addition, if the patients or guardians refuse to proceed during follow up postoperatively, study will be immediately stopped and the patients will be excluded.

**Data Management and Statistical Analysis**

Data sheets with coded identification of patients will be stored in the data storage of the department with automatic door locking system. Electronic data will be stored in personal computer with limited access by personal identification number. This will be reviewed and monitored by the monitoring committee of our institute.

Continuous variable will be analyzed with student’s t-test or Mann-Whitney U test as appropriate. Categorical variables will be evaluated using chi-square tests or Fisher’s exact tests. P<0.05 will be considered as statistically significant. Analysis will be performed with SPSS version 18.0 (SPSS Inc., Chicago, IL, USA)

**Quality Assurance**

The study protocol was reviewed thoroughly by IRB before approval and will be occasionally monitored by monitoring committee of our institute without notification. In addition, the principal

**Expected Outcomes of the Study**

We expected that propofol would reduce the inflammatory reaction and oxidative stress, which would modify the standard anesthesia care during RALRP.

**Dissemination of the Results and Publication Policy**

The result would change the standard anesthesia care during RALRP, and possibly influence the patients’ outcome after RALRP.

Publication will be led by the principal researcher Dr Han and Dr Roh by exploring the possible journals fitted for our research.

**Duration of the Project**

Data collection: 18 months after IRB approval

Analysis: 19-20 months after IRB approval

Writing and Revision: 21-24 month after IRB approval

**Problems Anticipated**

There will be no significant problem anticipated.

**Project Management**

GUR and DWH collected data.

GUR and DWH wrote the main manuscript text.

YS and DWH reviewed the initial manuscript and revised it.

JP and YMK contributed to the statistical analysis and table.

DWH prepared the figure and is the corresponding author.

All authors reviewed the manuscript.

**Ethics**

The day before surgery, the researcher meets the patients in a private room. The researcher explains the protocol in detail, ask and answer the questions. After a few hours, the researcher revisits the patient and asks him to sign the consent form if he is willing to participate. And the researcher notifies the patients that he can withdraw his will any time during the study and it would never affect his medical care.

The anesthetic agents used in this study are commonly used clinical anesthetic practice and the incidence of side effects or complication during this study will be similar to other clinical anesthetic practices in this institution. There will be no difference in anesthetic technique between study patients and others and there will be no need to report additional risk or safety issue related to this study. However, if there are any unpredictable events related to anesthesia occurred during study, the study will be immediately stopped and the detail will be reported to IRB.

**Informed Consent Forms**

The files were uploaded separately.

**References**

1. Reynolds W Jr. The first laparoscopic cholecystectomy. JSLS. 2001; 5: 89-94.

2. Wellwood J, Sculpher MJ, Stoker D, Nicholls GJ, Geddes C, Whitehead A, Singh R, Spiegelhalter D. Randomised controlled trial of laparoscopic versus open mesh repair for inguinal hernia: outcome and cost. BMJ. 1998; 317: 103-10.

3. Zacks SL, Sandler RS, Rutledge R, Brown RS Jr. A population-based cohort study comparing laparoscopic cholecystectomy and open cholecystectomy. Am J Gastroenterol. 2002; 97: 334-40.

4. Abassi Z, Bishara B, Karram T, Khatib S, Winaver J, Hoffman A. Adverse effects of pneumoperitoneum on renal function: involvement of theendothelin and nitric oxide systems. Am J Physiol Regul Integr Comp Physiol. 2008; 294: R842-50.

5. Nickkholgh A, Barro-Bejarano M, Liang R, Zorn M, Mehrabi A, Gebhard MM, Buchler MW, Gutt CN, Schemmer P. Signs of reperfusion injury following CO2 pneumoperitoneum: an in vivo microscopy study. Surg Endosc. 2008; 22: 122-8.

6. McLaughlin JG, Scheeres DE, Dean RJ, Bonnell BW. The adverse hemodynamic effects of laparoscopic cholecystectomy. Surg Endosc. 1995; 9: 121-4.

7. Safran D, Sgambati S, Orlando R 3rd. Laparoscopy in high-risk cardiac patients. Surg Gynecol Obstet. 1993; 176: 548-54.

8. Altintas F, Tunali Y, Bozkurt P, Kaya G, Uygun N, Aricioğlu F, Hacibekiroğlu M. An experimental study on the relationship of intra-abdominal pressure and renal ischemia. Middle East J Anesthesiol. 2001; 16: 55-66.

9. Mittal A, Phillips AR, Loveday B, Windsor JA. The potential role for xanthine oxidase inhibition in major intra-abdominal surgery. World J Surg. 2008; 32: 288-95.

10. Berkowitz DE, White R, Li D, Minhas KM, Cernetich A, Kim S, Burke S, Shoukas AA, Nyhan D, Champion HC, Hare JM. Arginase reciprocally regulates nitric oxide synthase activity and contributes to endothelial dysfunction in aging blood vessels. Circulation. 2003; 108: 2000-6.

11. Kaya Y, Coskun T, Demir MA, Var A, Ozsoy Y, Aydemir EO. Abdominal insufflation-deflation injury in small intestine in rabbits. Eur J Surg. 2002; 168: 410-7.

12. Guler C, Samli M, Aksoy Y, Demirbas M, Kilinç A, Ellidokuz E, Dincel C. Effects of carbon dioxide pneumoretroperitoneum on free radical formation in remote organs and use of verapamil as an antioxidant. J Endourol. 2004; 18: 245-9.

13. Ali NA, Eubanks WS, Stamler JS, Gow AJ, Lagoo-Deenadayalan SA, Villegas L, El-Moalem HE, Reynolds JD. A method to attenuate pneumoperitoneum-induced reductions in splanchnic blood flow. Ann Surg. 2005; 241: 256-61.

14. Ozmen MM, Zulfikaroglu B, Col C, Cinel I, Isman FK, Cinel L, Besler TH. Effect of increased abdominal pressure on cytokines (IL1 beta, IL6, TNFalpha), C-reactive protein (CRP), free radicals (NO, MDA), and histology. Surg Laparosc Endosc Percutan Tech. 2009; 19: 142-7.

15. Bachetti T, Comini L, Francolini G, Bastianon D, Valetti B, Cadei M, Grigolato P, Suzuki H, Finazzi D, Albertini A, Curello S, Ferrari R. Arginase pathway in human endothelial cells in pathophysiological conditions. J Mol Cell Cardiol. 2004; 37: 515-23.

16. Harris KF, Matthews KA. Interactions between autonomic nervous system activity and endothelial function: a model for the development of cardiovascular disease. Psychosom Med. 2004; 66: 153-64.

17. Runzer TD, Ansley DM, Godin DV, Chambers GK. Tissue antioxidant capacity during anesthesia: propofol enhances in vivo red cell and tissue antioxidant capacity in a rat model. Anesth Analg. 2002; 94: 89-93.

18. De La Cruz JP, Sedeno G, Carmona JA, Sanchez de la Cuesta F. The in vitro effects of propofol on tissular oxidative stress in the rat. Anesth Analg. 1998; 87: 1141-6.

19. Taniguchi T, Kanakura H, Yamamoto K. Effects of posttreatment with propofol on mortality and cytokine responses to endotoxin-induced shock in rats. Crit Care Med. 2002; 30: 904-7.

20. Rodriguez-Lopez JM, Sanchez-Conde P, Lozano FS, Nicolas JL, Garcia-Criado FJ, Cascajo C, Muriel C. Laboratory investigation: effects of propofol on the systemic inflammatory response during aortic surgery. Can J Anaesth. 2006; 53: 701-10.

21. Sanchez-Conde P, Rodriguez-Lopez JM, Nicolas JL, Lozano FS, Garcia-Criado FJ, Cascajo C, Gonzalez-Sarmiento R, Muriel C. The comparative abilities of propofol and sevoflurane to modulate inflammation and oxidative stress in the kidney after aortic cross-clamping. Anesth Analg. 2008; 106: 371-8.

22. Zhou W, Fontenot HJ, Liu S, Kennedy RH. Modulation of cardiac calcium channels by propofol. Anesthesiology. 1997; 86: 670-5.

23. Jordan JE, Zhao ZQ, Vinten-Johansen J. The role of neutrophils in myocardial ischemia-reperfusion injury. Cardiovasc Res. 1999; 43: 860-78.

24. Corcoran TB, Engel A, Sakamoto H, O'Shea A, O'Callaghan-Enright S, Shorten GD. The effects of propofol on neutrophil function, lipid peroxidation and inflammatory response during elective coronary artery bypass grafting in patients with impaired ventricular function. Br J Anaesth. 2006; 97: 825-31.

25. Corcoran TB, Engel A, Sakamoto H, O'Shea A, O'Callaghan-Enright S, Shorten GD. The effects of propofol on lipid peroxidation and inflammatory response in elective coronary artery bypass grafting. J Cardiothorac Vasc Anesth. 2004; 18: 592-604.

**Budget**

-Griess Reagent kit (NO): 2,000,000 KRW

-Nitric oxide Reagent: 2,169,000 KRW

-CRP: 2,397,000 KRW

-IL-6: 3,217,000 KRW

-TNF-α: 3,217,000 KRW

🡪 Total 13,000,000 KRW

**Other support for the Project**

Funding information: This study was supported by a faculty research grant of Yonsei University College of Medicine for 2013 (6-2013-0098)

**Collaboration with other scientists or research institutions**

none

**Links to other projects**

none

**Curriculum Vitae of investigators**

The files are uploaded separately.

**Other research activities of the investigator:** Dr Han’s current studies are following.

1. Predictive modeling of patient-controlled analgesia effect on postoperative pain management

2. Pharmacodynamic modeling of rSO_2_ during graft replacement of aorta

3. The effect of remifentanil and midazolam on ED95 of propofol for loss of consciousness in elderly patients

4. Validation of the Phase lag entropy (PLE) as an indicator of sedation in patients undergoing spinal anesthesia

5. Effect of dexmedetomidine on lung injury after subarachnoid haemorrhage in a rat

6. The relationship between the level of albumin and patient’s outcome in patients undergoing cytoreductive surgery combined with hyperthermic intraperitoneal chemotherapy (HIPEC).

7. A Randomized, multi-center, parallel group, active comparator, single blind, phase III clinical trial to evaluate the efficacy and safety of HNP-2001 (remimazolam) in surgical patients undergoing general anesthesia

**Financing and Insurance**

none
